# Estimates of present and future flood risk in the conterminous United States

**DOI:** 10.1088/1748-9326/aaac65

**Published:** 2018-02-28

**Authors:** Oliver E J Wing, Paul D Bates, Andrew M Smith, Christopher C Sampson, Kris A Johnson, Joseph Fargione, Philip Morefield

**Affiliations:** 1 School of Geographical Sciences, University of Bristol, Bristol, BS8 1SS, United Kingdom; 2 Fathom, Engine Shed, Station Approach, Bristol, BS1 6QH, United Kingdom; 3 The Nature Conservancy, 1101 West River Parkway Suite 200, Minneapolis, MN 55415, United States of America; 4 US Environmental Protection Agency, National Center for Environmental Assessment, 1200 Pennsylvania Avenue, NW Washington, DC 20460, United States of America

**Keywords:** flood risk, large-scale flood models, flooding, USA

## Abstract

Past attempts to estimate rainfall-driven flood risk across the US either have incomplete coverage, coarse resolution or use overly simplified models of the flooding process. In this paper, we use a new 30 m resolution model of the entire conterminous US with a 2D representation of flood physics to produce estimates of flood hazard, which match to within 90% accuracy the skill of local models built with detailed data. These flood depths are combined with exposure datasets of commensurate resolution to calculate current and future flood risk. Our data show that the total US population exposed to serious flooding is 2.6–3.1 times higher than previous estimates, and that nearly 41 million Americans live within the 1% annual exceedance probability floodplain (compared to only 13 million when calculated using FEMA flood maps). We find that population and GDP growth alone are expected to lead to significant future increases in exposure, and this change may be exacerbated in the future by climate change.

## Introduction

In 2016, global economic losses as a result of flooding totalled $56bn (all values are 2017 USD), with $10bn of this accounted for by the August floods in Mississippi and Louisiana alone [1]. In the US over the past 30 years, freshwater flooding has caused an average of $8.2bn in damages each year, though this average masks an upward trend in flood losses over time [2-4]. This is also the case globally, with the major driver thought to be the increased exposure of people and assets [5, 6].

The Federal Emergency Management Agency (FEMA) has produced maps delineating the Special Flood Hazard Area for nearly all current coastal flood hazard areas in the US, and rigorous estimates have been published indicating how many people are exposed and how exposure is distributed nationally [7, 8]. Maps delineating fluvial (riverine) and pluvial (rainfall-driven) flooding, however, are only partially complete nationwide, and no comprehensive estimate of US population exposure currently exists. Where they are available, FEMA flood maps are of varying age and levels of quality. They also have notably poor coverage of smaller catchments, which is a trait shared by many of the hazard maps that are used to inform risk calculations at global or continental scales. For example, the framework for flood risk assessments set out by Winsemius *et al* [9] which is the current state-of-the-art in large-scale flood risk analytics [10-13], excludes rivers below Strahler [14] order 6 (catchments smaller than roughly 10 000 km^2^). This means that risk generated by these smaller streams, which may be situated in or around residential or commercial areas, is not captured. Further, coarse-resolution terrain data and the simplistic representation of the physics of flood spreading are characteristics shared by a majority of existing large-scale models [15]. It is evident, therefore, that previous large-scale efforts to quantify flood exposure (in terms of population and economic assets) and risk (in terms of deaths and economic losses) have known limitations that will lead to misestimation of these quantities (see supplementary section 1 available at stacks.iop.org/ERL/13/034023/mmedia for an explanation of the terminology used here).

This study presents new estimates of current and potential future flood exposure and risk using high-resolution hazard, population, asset and projected development maps of the entire conterminous United States (CONUS). These layers are of significantly higher quality and spatial coverage than those that have previously informed exposure and risk estimations. Validation of the new hazard layers [16] suggests they are of commensurate quality to local studies carried out by US government agencies. These new high-resolution analyses with a realistic representation of flood physics indicate that the population exposed to serious flooding in CONUS is 2.6–3.1 times higher than previous estimates. This has major consequences for flood risk management and policy in the US.

## Methods

Flood hazard maps representing both fluvial (river flooding, in catchments larger than 50 km^2^) and pluvial hazard (flooding from intense rainfall directly onto the land surface, simulated in river catchments of all sizes) are derived using return period discharges and rainfalls from regionalised flood frequency analyses [17] as inputs to a computationally efficient flood inundation model based on an inertial formulation of the shallow water equations in two dimensions [18, 19]. The underlying digital elevation model (DEM) is sourced from the US Geological Survey National Elevation Dataset, with simulations run at the native DEM resolution of 1″ (~30 m). The National Levee Database produced by the US Army Corps of Engineers (USACE) is incorporated explicitly into the model in order to represent known flood defences. Further information on the flood hazard model can be found in supplementary section 2.1 and Wing *et al* [16]. The explicit incorporation of flood defences; higher vertical accuracy and finer horizontal resolution of terrain data; better representation of fluid physics; and coverage of all basin scales all represent step changes over previous large-scale hazard analyses [9].

**Figure 1.**
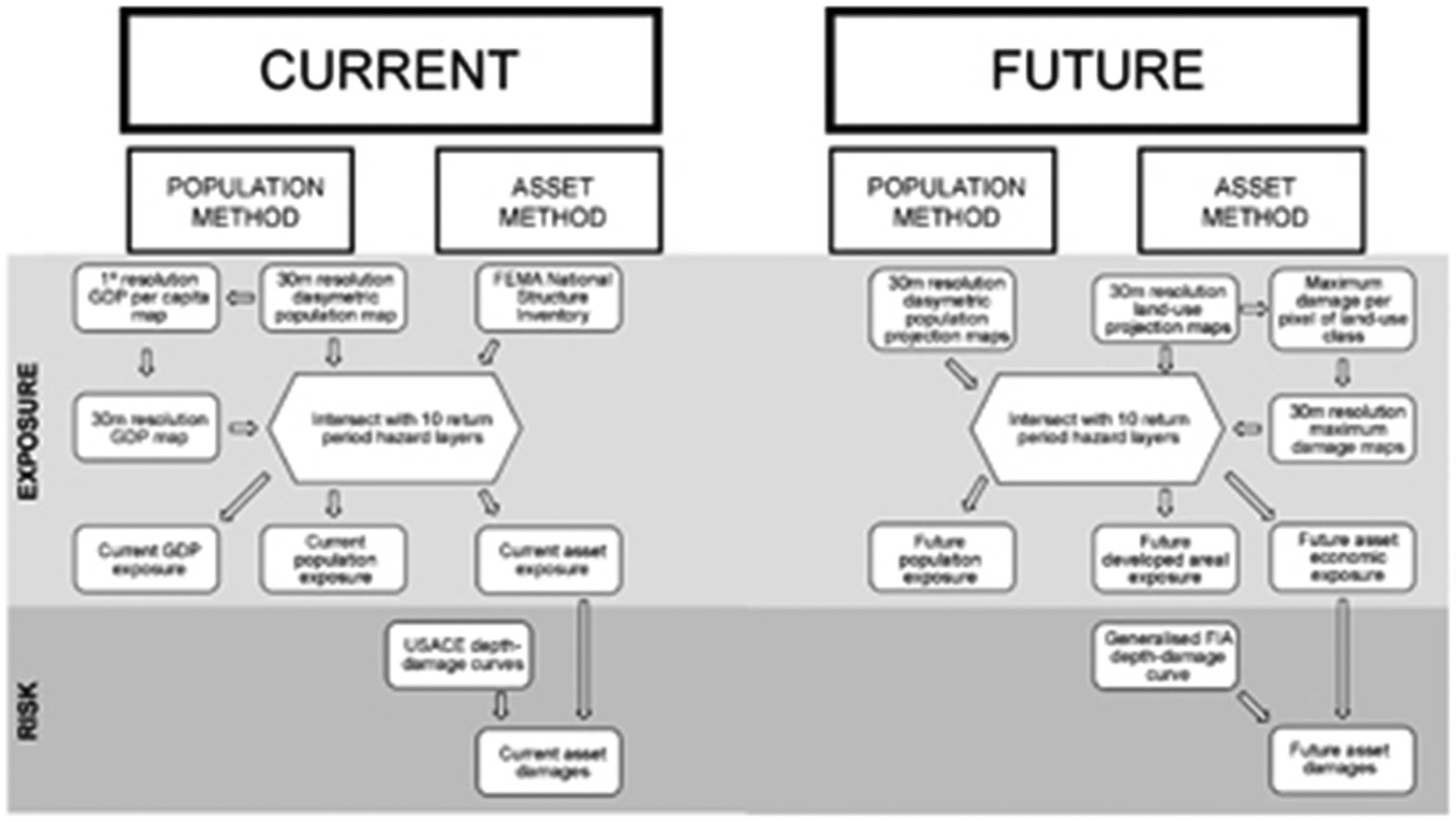
Schematic diagram of the methodology for generating exposure and risk estimates. FIA stands for Federal Insurance Agency.

Various high-resolution datasets were employed to translate hazard into exposure and risk for both current and future conditions (see supplementary section 1). Current distributions of people and assets are detailed by the US Environmental Protection Agency (USEPA) population density map and the FEMA National Structure Inventory. Their projected distribution under different future Shared Socio-economic Pathway (SSP) scenarios has been produced by the USEPA Integrated Climate and Land-Use Scenarios (ICLUS) project [20]. These data are also a significant advance on the more aggregated datasets that have informed previous calculations. The methodology, detailed schematically in figure 1, is explained more fully in supplementary section 2.

## Results

**Figure 2.**
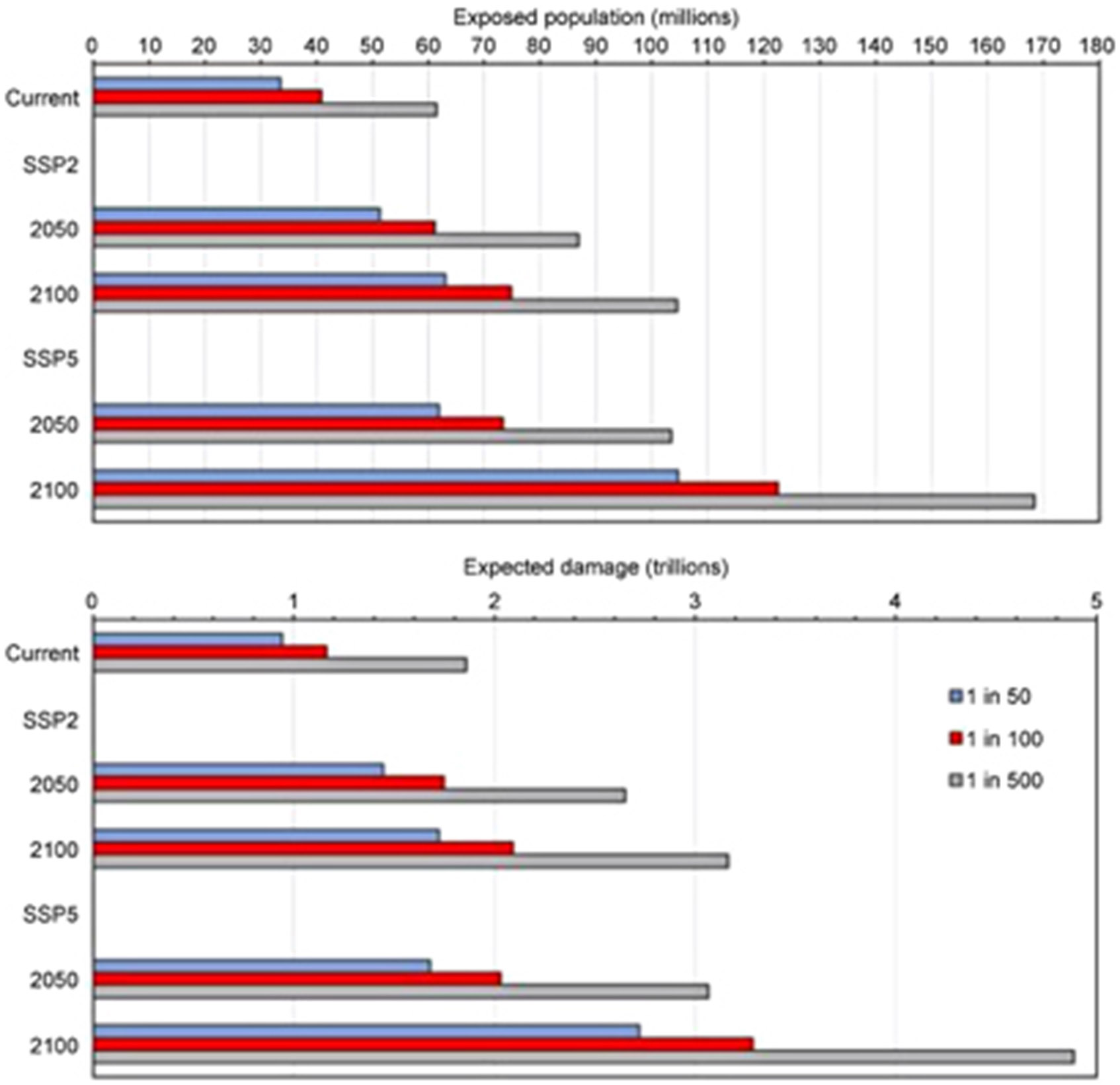
Selected exposure (population) and risk (damage) estimates for present and future 1 in 50-, 100- and 500 year floods. SSP2 represents a medium growth scenario, in terms of population and development, while SSP5 represents a higher one.

**Figure 3.**
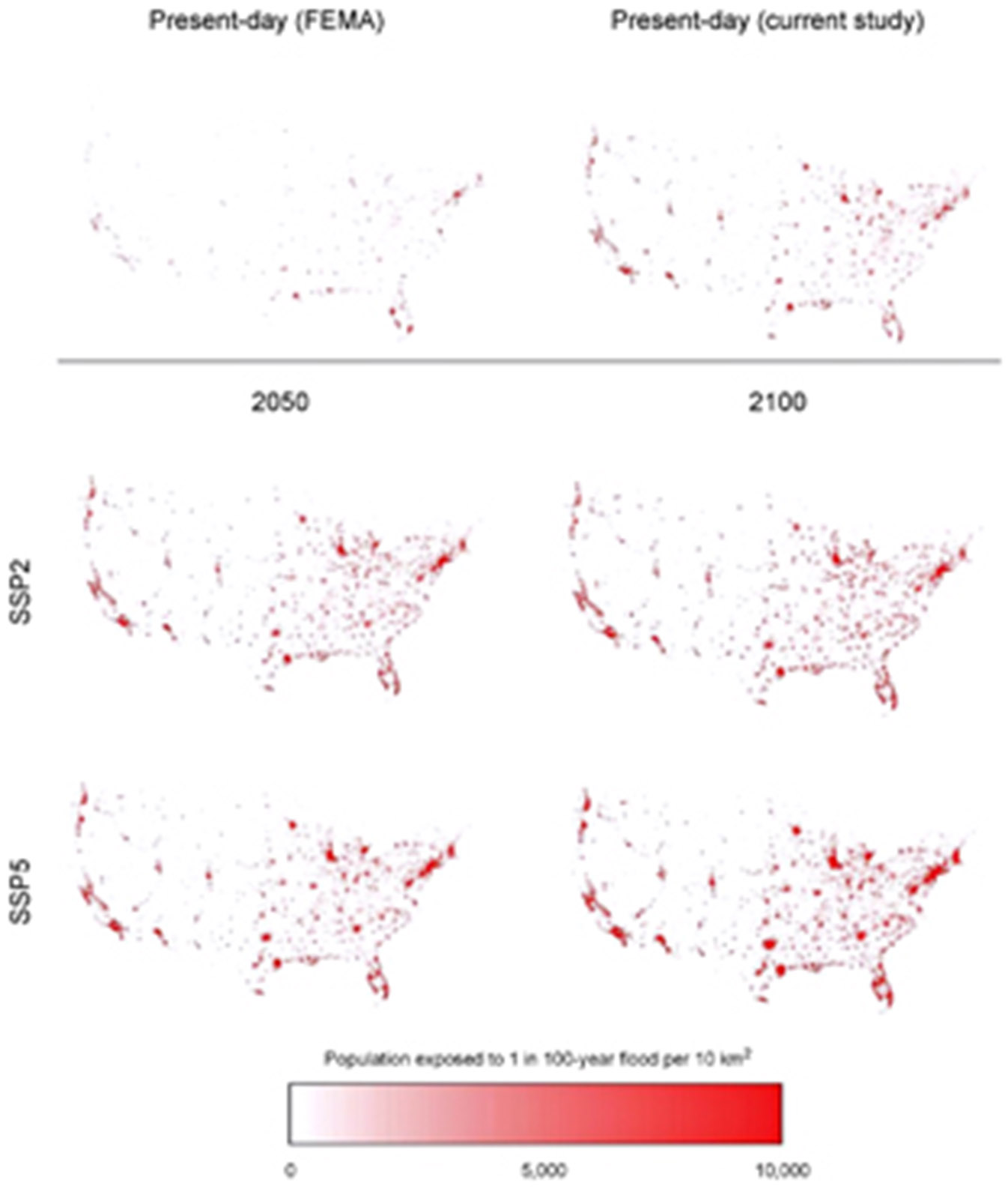
Distribution of population exposed to a 1 in 100 year flood across the CONUS for the present and future.

Our updated and refined estimates for rainfall and river-flow driven flood exposure and risk in the CONUS for both current and future conditions supersede previous estimates provided by patchy local models and poorer quality global ones. Estimates described as ‘current’ or ‘present-day’ are derived from exposure data describing socio-economic conditions in 2010. The analysis shows that 40.8 million people (13.3% of the population) are currently exposed to a 1 in 100 year (1% annual exceedance probability) fluvial or pluvial flood in the CONUS, which translates to a GDP exposure of $2.9 trillion (15.3% of total GDP). This represents substantially higher exposure than previous estimates suggest. The World Resources Institute Aqueduct Global Flood Analyzer [9, 13, 21] (hereafter, Aqueduct) suggests that 15.7 million people and $0.7 trillion of GDP are exposed to a 1 in 100 year flood in the US. FEMA flood maps, intersected with the population data used in this study, indicate 13.0 million people are exposed. Figure 3 elucidates where the differences between our analysis and FEMA exposures arise. While our study identifies exposure missed by FEMA across the country, higher concentrations of newly identified exposure are particularly evident along the Pacific coast, in urban centres around the Great Lakes and across inland western US. With no coverage of small rivers in the Aqueduct data and incomplete coverage in the FEMA flood maps, it is not surprising that our study has identified additional areas of flood exposure. This analysis indicates that previous estimates capture roughly one-third of the exposure identified in our 1 in 100 year floodplain. A more detailed discussion of our results can be found in supplementary section 3. Figure 2 and supplementary table 1 detail these current population-based exposure estimates further.

**Figure 4.**
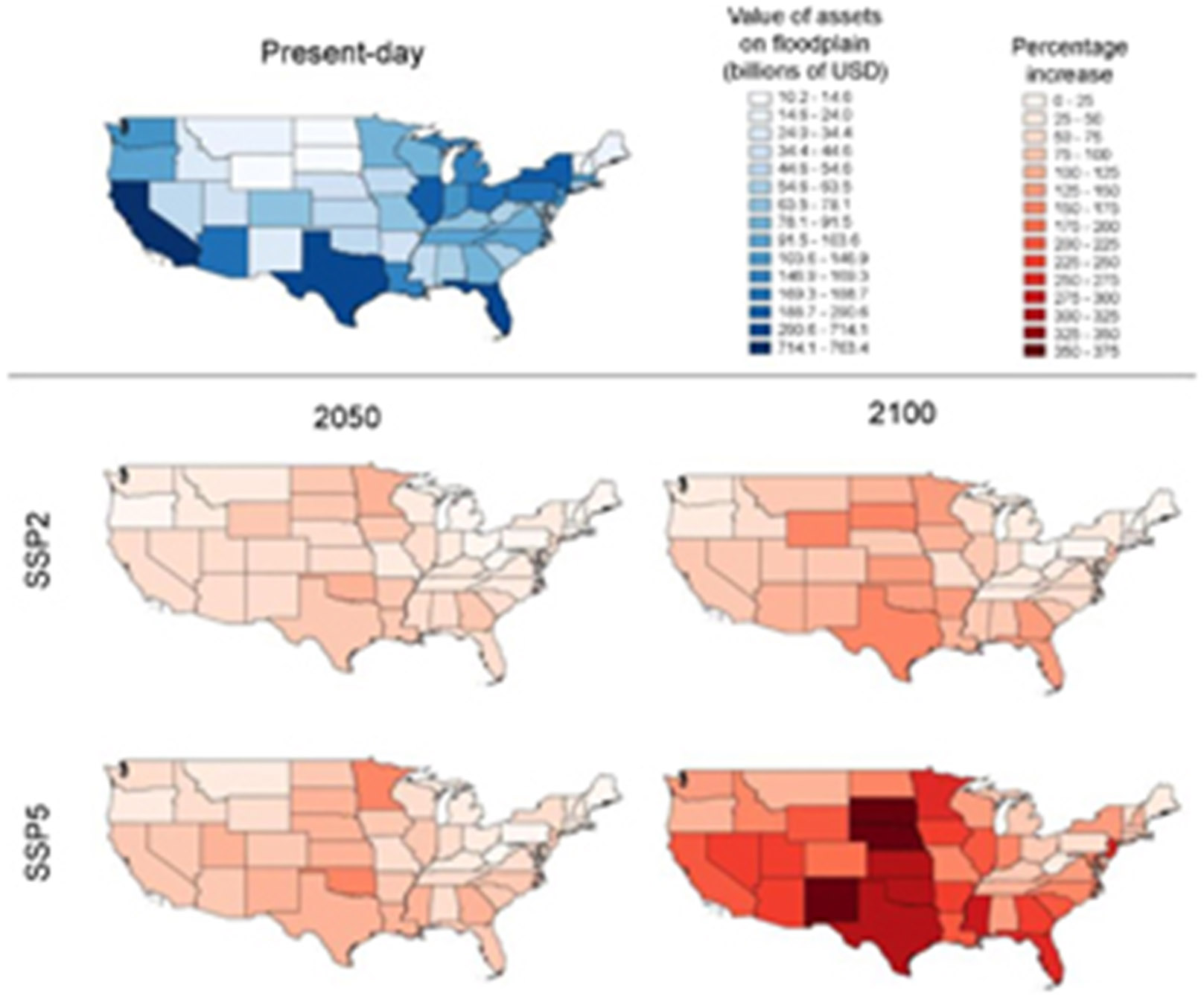
Maps depicting the value of assets within the 1 in 100 year floodplain, split by state. The ‘current’ map (blue) indicates the absolute value of assets within each state’s floodplain. The ‘future’ maps (red) indicate the proportional increase in exposed assets from the present-day to the respective year under a particular scenario.

The total value of assets within the present-day CONUS 1 in 100 year floodplain is $5.5 trillion, with $1.2 trillion of this at potential risk from flood damage. Further details on all asset-based flood exposure and risk can be found in supplementary table 2. Supplementary figure 2 indicates that Louisiana, Florida, Arizona and West Virginia are particularly over-exposed, with 32%, 28%, 26% and 25% of their total asset values situated within the 1 in 100 year floodplain respectively. From figure 4, it is evident that the absolute value of assets on the Floridian floodplain is also particularly high at $714 billion: Florida is thus a hotspot of flood exposure. While their percentage of exposed assets are not particularly high, California and Texas have high absolute values of flood exposure at $763 billion and $400 billion respectively.

In contrast to the comparison between our analysis and Aqueduct data for population exposure, our asset damage estimates are smaller than those of the Aqueduct data. Aqueduct estimates that $3.3 trillion of assets are at risk of damage from the 1 in 100 year flood, over double the corresponding value in our analysis. Perhaps surprisingly, with a smaller spatial flood extent, the Aqueduct hazard map translates to a larger estimate of flood risk. We hypothesize that the coarse land-use maps used by Aqueduct do not capture the nuances of where assets are situated in reality. The 5′ (~10 km) grid cells, which indicate percentage urban area, used in Aqueduct to drive exposure estimates, are unlikely to be sufficiently resolved to capture the true distribution of assets. In reality, buildings will generally be more concentrated outside the floodplain than within it, but this is not captured by 5′ grid cells. In addition, differences may arise from the depth-damage curves used and asset values assigned.

The New York University (NYU) Furman Center has merged FEMA flood maps with census block-level housing information, determining the number of housing units within the floodplain [22]. The NYU data indicate that 6.8 million housing units fall within the 1 in 100 year floodplain, while our analysis finds that 15.4 million houses are situated there. This illustrates the inadequacies of using incomplete hazard maps (from FEMA) in combination with aggregated socio-economic data (from the US Census Bureau) for flood exposure estimates: the NYU methodology failed to capture even half of the properties on the floodplain identified by our method.

Future population exposure is also detailed in figure 2. The general trends are perhaps as expected, with greater exposure by 2100 and greater exposure increases in high growth scenarios (e.g. SSP5 vs. SSP2). Absolute future increases in population exposure are naturally higher for larger return periods: an increase of 17.8 million for the 1 in 50 year flood vs. 25.4 million for the 1 in 500 year flood in 2050 under the SSP2 scenario. Percentage future increases confer something more interesting: for the same scenario and year, the 1 in 50 year population exposure increases by 53% while the 1 in 500 year is 41%. This means that more frequently inundated areas are experiencing faster population growth than less frequently inundated ones. In other words, the more hazardous areas in the floodplain (1 in 50 year zone) are projected to experience a higher population growth rate than the floodplain as a whole (1 in 500 year zone). Supplementary table 3 indicates the proportion of the population within each floodplain, where it is apparent that a larger percentage of the total CONUS population will reside in a floodplain in the future. Present-day 1 in 100 year flood exposure stands at 13.3%, but increases to 15.6%–15.8% in 2050 and 16.4%–16.8% in 2100. It is evident, therefore, that an increased share of future development is projected to take place within the floodplain. The SSP5 scenario places slightly more people on the floodplain as a proportion of total population than SSP2, but the lower-growth SSP2 scenario still projects a proportional increase in population exposure. Figure 3 highlights that this increased exposure is generated across the CONUS as population increases. In particular, the expansion of urban areas around the Great Lakes, Florida coast, northeast US and Texas see substantial increases in total population exposure.

Future trends in asset flood risk are similar to the population-based estimates (figure 2 and supplementary table 4). Notably, the total area of developed land within the 1 in 100 year floodplain in 2100 under the SSP5 scenario is roughly equivalent to the land area of Colorado. This equates to a projected value of exposed assets roughly equivalent to the current GDP of the US. Figure 4 indicates where asset exposure increases are concentrated. In 2050, Minnesota and the Great Plains see substantial proportional increases in assets within the floodplain. In particular, exposed assets at least double by 2050 in Oklahoma. Currently, many of these states are relatively undeveloped, so a large increase in the percentage flood exposure does not necessarily amount to a high absolute increase. California, with $763 billion worth of assets on the floodplain already, sees a 50%–100% increase in exposure by 2050. These patterns are even more pronounced by 2100. The currently less-developed Great Plains still experience the greatest proportional increase in exposure, with increases in South Dakota, Nebraska and New Mexico of almost five-fold in relation to the present-day. California, Florida and Texas, already with high absolute exposure, will see within-floodplain asset values triple or quadruple by 2100 under the SSP5 scenario. Interestingly, West Virginia, which is currently proportionally highly exposed with respect to other states (supplementary figure 2), sees very little change in its exposure going into the future. Even under the SSP5 scenario in 2100, the value of assets on West Virginia’s 100 year floodplain only increases by one-quarter. New England experiences relatively little increase in asset exposure under both scenarios analysed.

The estimates produced in this study are subject to various sources of uncertainty, which are expanded on below and in supplementary section 5. The USACE National Levee Database is incomplete, meaning that some of the areas that we have identified as most at risk may be ‘false alarms’ where people and assets are actually defended from flooding. A comprehensive catalogue of flood defences in the US should be the focus of future research to ameliorate this issue. The effect of climate change is not incorporated into future flood hazard projections as there is not yet compelling evidence of a climate change signal in the observed losses of fluvial and pluvial flooding, although there is a scarcity of gauging data with which to test this [23]. This poor understanding of climate change effects on flooding at local scales therefore means the Intergovernmental Panel on Climate Change has low confidence in future projections of flood hazard [23]. It does stand to reason, however, that an enhancement of the hydrological cycle in a warming world will lead to increased rainfall and greater flood hazard in some regions of the US [24]. Where studies have sought to quantify this, flood frequency response in the US is mixed for all future scenarios: some areas are projected to experience increased flood hazard, while others will see it reduced [10, 24]. Predicting socio-economic changes throughout the 21st Century is also a difficult undertaking, and is naturally subject to much uncertainty. Out of a wide range of possibilities, two scenarios are used here: a ‘medium’ case (SSP2) which closely tracks the national US Census projection through 2060, and a ‘high growth’ case (SSP5) which has the US population growing to over 700 million people by 2100 [20]. These USEPA ICLUS projections should be seen purely as a set of plausible future conditions developed using different assumptions.

## Conclusions

We present the most spatially detailed flood exposure and risk estimates, both present and future, of the CONUS to date. Our analysis shows that both FEMA flood maps and previous large-scale risk estimates likely significantly underestimate population exposure, while the latter simultaneously overestimates flood risk. This study is a first of its kind; utilising highly resolved, spatially comprehensive flood hazard information derived from a model that properly represents the physics of flood spreading in combination with high-resolution estimates of the present and future distribution of people and assets. With this detailed spatial information on present-day flood risk, federal and state agencies can take appropriate action to mitigate losses [26]. Use of USEPA population and land-use projections means that particular attention can be paid to floodplains where development is projected. Steps to conserve such areas or ensure adequate defences are in place could avoid the exposure of trillions of dollars of assets, not to mention the human suffering caused by loss of property and life.

## Supplementary Material

Supplement1
