# Supplementary material for: Estimates of present and future flood risk in the conterminous United States
Source: Environ Res Lett. Author manuscript; Available in PMC 2025 Apr 8. (PMC11977400; doi:10.1088/1748-9326/aaac65)
Supplement: Supplement1 [file NIHMS2056067-supplement-Supplement1.docx]

**SUPPLEMENTARY INFORMATION**

**Estimates of present and future flood risk in the conterminous United States**

Oliver E. J. Wing, Paul D. Bates, Andrew M. Smith, Christopher C. Sampson, Kris A. Johnson, Joe Fargione, Philip Morefield

**Table of Contents**

1 Terminology.......................................................................................................................2

2 Methods.............................................................................................................................3

3 Detailed Results.................................................................................................................6

4 Validation...........................................................................................................................9

5 Limitations........................................................................................................................11

6 Supplementary Figures....................................................................................................14

7 Supplementary Tables.....................................................................................................17

8 Supplementary References.............................................................................................19

**1 Terminology**

Risk is defined in this paper as being the product of hazard, exposure and vulnerability; consistent with the definition of the United Nations International Strategy for Disaster Risk Reduction^1^. Hazard refers to the nature, magnitude and probability of the flood event itself. Socio-economic data, such as where people live or where buildings are situated, are intersected with the delineation of the hazardous area to generate flood exposure. This process is described schematically in the ‘exposure’ box (lighter grey) of Figure 1 in the main paper. Exposure doesn’t account for factors such as the damage a flood may cause to a structure, the number of fatalities arising from an event, or the propensity to mount a short-term response to a flood in order to mitigate its effects (e.g. an early warning system leading to the erection of temporary defences). These factors broadly relate to the vulnerability of an area, defined as the susceptibility of the exposed people or assets to experience loss (in various forms), which creates the distinction between flood exposure and flood risk. For example, exposure may be the total value of buildings within a flood zone, while risk may be the expected damage to these buildings. To constrain risk in these estimates, a relative depth-damage curve (representing vulnerability) is applied to the exposed assets (see the ‘risk’ box (darker grey) of Figure 1 in the main paper). This relationship determines the economic damage a certain depth of water causes to an asset as a percentage of its total value. While this generates some idea of flood risk, it still does not account for the wealth of local-level factors which are ultimately required for a more accurate estimation. On top of this, the depth-damage relationships themselves are subject to much uncertainty^2^. This and other uncertainty is explored in Supplementary Section 5, but the key point here is that uncertainty accumulates when moving from a hazard to a risk calculation. Observing the hazard layers in isolation is already subject to much uncertainty; but a risk estimation is not only subject to uncertainty in the hazard layer, but also to uncertainty in asset values, the location of assets and the economic effect of flooding on a certain asset. Although a risk estimation may seem to provide something more tangible and interesting (e.g. expected damage from a flood event), an exposure estimation (e.g. total value of assets within a certain floodplain) will be subject to less uncertainty, and a hazard estimation (e.g. total area of the floodplain) to even less than that. The data and methodology employed to generate hazard, exposure and, ultimately, risk estimations are detailed in the following section.

**2 Methods**

**2.1 Hazard**

The hazard layers employed to inform these estimates represent both fluvial (flooding from rivers) and pluvial (flash-flooding arising from direct rainfall onto the land surface) perils and are detailed more fully in Wing *et al.*^3^.

The fluvial model component is driven by design discharges of ten different recurrence intervals between a 1 in 5-year (20% annual probability) and 1 in 1000-year (0.1% annual probability). These are generated using a global regionalised flood frequency analysis (RFFA)^4^. This overcomes the issue of spatial sparsity in river gauges by transferring flow data from a gauged basin to a similar ungauged basin. It assumes that catchments with similar characteristics, such as climatology, upstream annual rainfall and land area, will have a similar flood frequency. Suitably homogenous groups are assigned a flood estimation index, permitting the ten return period discharges to be generated using the mean annual flood and flood frequency curve of each river reach. The US Geological Survey (USGS) National Elevation Dataset (NED) is used to generate the digital elevation model (DEM) at 1 arc second (~30m) resolution, and simulations are executed at this high resolution. River channels are delineated by the HydroSHEDS global hydrography dataset^5^ and, if they are narrower than 1 arc second, are represented subgrid^6^. The RFFA-generated discharges are routed by means of an efficient inertial formulation of the shallow water equations in two dimensions, based on LISFLOOD-FP^6,7^, through the channels and over the floodplain. Flood defences are incorporated explicitly into the model, sourced from the US Army Corps of Engineers (USACE) National Levee Database (NLD).

The fluvial component is only executed for catchments larger than 50 km^2^. Smaller catchments are highly heterogenous in terms of their flood frequency response which, alongside a lack of stream records for such areas, renders them unsuitable to be incorporated into the RFFA. Instead, these areas are simulated by the pluvial model, owing to their ‘flashy’ flood response to intense local rainfall events. National Oceanic and Atmospheric Administration (NOAA) Intensity-Duration-Frequency curves form the basis of rainfall scenarios generated for this component. In recognition that not all of this rainfall will generate flood hazard, an infiltration equation is applied^8^ depending on the underlying soil type (sourced from the Harmonized World Soil Database) and, in urban areas^9^, a certain drainage design standard is assumed.

As alluded to in the main paper, the Intergovernmental Panel on Climate Change has low confidence in even the direction of change in future flooding^10^. This significant uncertainty is emergent in current climate models, which have a low level of agreement with regards to changes in flood-inducing rainfall. The propagation of modelled precipitation through rainfall-runoff models further amplifies these uncertainties. Smith *et al.*^11^ demonstrated that the estimation of present-day extreme flow discharge in a well-gauged catchment already presents a significant challenge. Indeed, the study suggested that the incorporation of future flood projections only led to a small increase in the uncertainty that was already present in the extreme flow estimation procedures for current conditions. Given that these conclusions were reached using a well-calibrated rainfall-runoff model in a gauged catchment with a long historic record, the it is likely that the scale of current uncertainties in extreme flow estimation compared to potential climate change effects will be even greater for less well understood or ungauged catchments. Therefore, the hazard layers used to estimate future flood risk are the same as those used for current estimates.

**2.2 Current Flood Exposure and Risk**

**2.2.1 Population Exposure**

To estimate the total number of people exposed to floods of various magnitude, the hazard layers are intersected with a US Environmental Protection Agency (USEPA) map of population density, which distributes 2010 census block population counts to 30m pixels based on land-use and slope. It is also combined with a GDP per capita map derived from G-Econ, which represents the heterogeneity of GDP distribution across the USA. This provides a monetary, as well as popular, value of current flood exposure.

**2.2.2 Asset Exposure and Risk**

The FEMA National Structure Inventory (NSI) is used to estimate the value of assets currently exposed to fluvial and pluvial flooding in the CONUS. The NSI details the nature, value and location of buildings and their contents in the United States. Upon intersection with the hazard layers (to generate exposure), a specific USACE depth-damage function based on building type is applied to calculate expected damages from a certain return period flood event (risk). The National Land-Use Dataset (NLUD)^12^ is used to calculate the area of exposed developments.

**2.3 Future Flood Exposure and Risk**

Future flood exposure and risk is estimated using population and land-use projections from the USEPA Integrated Climate and Land-Use Scenarios (ICLUS) project. These projections were generated using inputs and assumptions corresponding to Shared Socioeconomic Pathways (SSPs), which describe broad societal changes such as human migration patterns, fertility rates and technological innovation. Projections under SSP2 (medium population growth, historical migration patterns continue) and SSP5 (high population growth, medium-sized cities see increased in-migration) are used for the years 2050 and 2100. The projections are at 90m resolution, but are resampled to 30m in order to align with the hazard layers. A fuller explanation of ICLUS can be found in the USEPA report^13^.

**2.3.1 Population Exposure**

As with the current population-based exposure estimates, the hazard layers are intersected with the population projections to indicate the future population exposed to rainfall and river-flow driven flooding in 2050 and 2100.

**2.3.2 Asset Exposure and Risk**

The National Structure Inventory was used to calculate asset values per pixel of each ‘developed’ land-use class in the present-day NLUD. These values were then iterated across the CONUS for each ICLUS land-use projection and intersected with the hazard layers. The area and value of exposed developments are calculated, as well as the expected damage using a Federal Insurance Agency-derived generalised depth-damage relationship.

**3 Detailed Results**

This supplementary section builds on analysis detailed in the main paper and also draws on new discursive themes. Owing to the former, it should be viewed in conjunction with elements of the main text.

**3.1 Current Flood Exposure and Risk**

**3.1.1 Population Exposure**

Supplementary Table 1 details present-day population-based flood exposure estimates for 1 in 50-, 1 in 100- and 1 in 500-year events. As shown in Supplementary Fig. 1, these results well exceed leading contemporary exposure estimates. It is evident, therefore, how much exposure these current estimates are missing based on their incomplete spatial coverage. FEMA flood maps only cover around 61% of the CONUS land area, and even within this many small headwater catchments are un-modelled^3^. A growing body of research also highlights the inadequacies of FEMA data where it does exist. Blessing *et al.*^14^ compared FEMA flood maps to loss claims from five storms near Houston, Texas. Only one-quarter of the claims were located within the FEMA-delineated 1 in 100-year flood zone, despite none of the storms having a return period greater than a 1 in 50-year. This constitutes a dramatic underestimation of the floodplain which, alongside poor spatial coverage, means it is to be expected that consequent exposure calculations will also be underestimates.

As a typical global flood risk product we consider the Aqueduct dataset, which adopts the Winsemius *et al.*^15^ framework. This provides comparable estimates of population exposure to FEMA. Rather than emulating FEMA’s patchy coverage of smaller rivers, Aqueduct excludes all rivers with a Strahler^16^ order of less than 6 (typically basins below 10,000 km^2^). This is likely why both datasets produce broadly similar underestimates. It is also worth noting the similarity of results across all three return periods for the Aqueduct data. Trigg *et al.*^17^ observe that the underlying hazard layer of the Aqueduct data, GLOFRIS, displays relatively little sensitivity to the probability of the flood event in terms of total flooded area. This also appears to be the case in terms of population exposure: only 2.6 million more people are exposed to the 1 in 500-year flood compared to the 1 in 50-year. In reality, this is unlikely to be the case. Our analysis shows population exposure almost doubles from the 1 in 50- to the 1 in 500-year event.

**3.1.2 Asset Exposure and Risk**

Supplementary Table 2 presents the full suite of asset exposure and risk estimates for the present-day, while Supplementary Fig. 2 indicates which states are proportionally over- or under-exposed. For example, 32% of the total value of assets in Louisiana lie within the 1 in 100-year floodplain, while only 7% are exposed in Maryland.

**3.2 Future Flood Exposure and Risk**

Aqueduct modelled future flood risk scenarios in 2030, while our projections were in 2050 and 2100. In order to compare estimates, 2030 calculations are made by interpolating between our current and 2050 values. The selected Aqueduct figures are from the SSP2 socio-economic scenario for 2030 with no hydrological change to make the two datasets as comparable as possible.

**3.2.1 Population Exposure**

The number of people exposed to 1 in 50-, 100- and 500-year floods in the future is detailed in Supplementary Table 3. Differences between Aqueduct data and our analysis for future population exposure have a broadly similar theme to differences in present-day estimates. As shown in Supplementary Fig. 3, our estimate for the number of people exposed to a 1 in 100-year flood is almost triple the equivalent Aqueduct figure. Relative insensitivity of Aqueduct estimates to return period is also displayed once again.

**3.2.2 Asset Exposure and Risk**

Future asset exposure and risk estimates are detailed in Supplementary Table 4. In comparison to our data, Aqueduct simulates significantly higher flood risk in the CONUS (see Supplementary Fig. 4). This is consistent with the comparisons for present-day flood damage estimates shown in Figure 2 of the main paper.

**4 Validation**

The hazard layers, in isolation, have undergone validation against flood maps produced by FEMA and USGS in a previous study^3^. This work demonstrated that the continental-scale model used here captured around 90% of the floodplain delineated by these high quality flood maps and, in some instances, was a near perfect match. Validating elements of flood risk analyses that go beyond a specification of flood hazard is, however, a notoriously difficult task. The only tangible estimate produced in this study that can also be measured in reality for validation purposes is flood losses, which are themselves highly uncertain^18^. The NOAA National Weather Service has compiled annual losses as a result of freshwater (fluvial and pluvial) flooding for over a century. Their annual average loss (AAL) of the past 30 years amounts to $8.2 billion^19^.

In order to calculate an AAL from our analysis, an exceedance probability-impact curve must be constructed. AAL is simply the area under this curve, though it can be ‘truncated’ at a certain return period to indicate a flood defence standard^20^. In the US, river flood defences are typically built to withstand events with a return period of 1 in 100-years or greater, and storm sewer networks typically protect urban areas against rainfall flooding events with a return period of 1 in 10- or 1 in 20-years. Supplementary Fig. 5 illustrates the exceedance probability-impact curve for our simulations. Assuming floods with a return period lower than 1 in 100-year are not damaging, this analysis produces an AAL of $12.5 billion; somewhat aligning with NOAA observations. Under this strict assumption, it appears that our analysis has some skill in providing flood risk information. Relaxing this assumption to a more realistic 1 in 10-year nationwide defence standard (i.e. floods smaller than those with a 1 in 10-year return period cause no damage) generates an AAL of $77.5 billion. Reasons for the differences to the NOAA observed AAL of $8.2 billion could be numerous and are explored further below and in Supplementary Section 5.

It is apparent that this model framework has a positive bias towards low return period flood events, likely owing to deficient defence representation. The USACE National Levee Database is known to be incomplete for even large structural defences^21^, but also fails to catalogue more ‘informal’ berms and other small-scale structures that may unintentionally defend against high-frequency, low-magnitude flood events. Floods in these locations will cause relatively little damage in reality, but may inundate high risk areas in the model. All flood defences, large and small, will naturally be captured by the observed AAL. Similarly, the pluvial model component may not adequately represent the drainage capacity of urban areas, meaning too much of the rainfall during a storm is modelled as hazardous. It is encouraging that there is reasonable agreement between modelled and observed values with the exceedance probability-impact curve truncated at 1 in 100-years (most high-risk areas will be defended to this standard), and it suggests that more accurate estimates of lower return period events can be produced with improved defence representation. With that being said, it is likely that the NOAA observations are negatively biased towards both high and low return period flood events. By their own admission, damages are often underreported and, for small localised flood events, the information might not ever reach the National Weather Service at all. Additionally, it is implicit that the 30-year average will not pick up very low-probability, high-impact flood events, thus underestimating nationwide risk to these. Furthermore, our analysis has calculated loss at one moment in time (the present-day), whereas the NOAA 30-year average loss contains damages from a time when the US was less developed. In other words, a flood simulated by our model may have generated substantial losses in the present-day but not in the 1990s because of urban development over this period. It is therefore prudent not to overstate the verity of the NOAA observations: both sets of data, modelled and observed, are subject to error and provide different, and useful, information. For these reasons, we restrict the focus of our analysis to higher return period floods (1 in 50-years and above) as here we can be more confident that the model has reasonable predictive skill and that the conclusions we draw are robust.

Comparing the AAL derived from the Aqueduct model, a current state-of-the-art global flood risk framework, to NOAA observations produces a much starker deviation (see Supplementary Fig. 5). Assuming a 1 in 10-year defence standard, the AAL based on Aqueduct data comes to $249 billion. Only when truncating the curve between the 1 in 500- and 1 in 1000-year exceedance probabilities do the NOAA observations converge with an Aqueduct AAL.

**5 Limitations**

The flood exposure and risk estimations presented in this analysis are subject to several limitations; the major ones are outlined in this section. As mentioned in Supplementary Section 2, some estimates are more uncertain than others (e.g. flooded area is less uncertain than expected damage). This section is structured in such a way that limitations are examined separately where they pertain to hazard, exposure or risk. Once again, we reiterate that these limitations are cumulative, in that the final risk estimation contends with uncertainty in hazard and exposure estimations also. It is also worth noting that all future estimates are significantly more uncertain than those for the present-day. The uncertainties discussed in this section do not invalidate the analyses presented here, instead they should be seen as the foci of future research to continue advancements in this field.

**5.1 Uncertainty in hazard estimation**

The underlying terrain data, the USGS NED, has a relative mean point-to-point accuracy of roughly 27 cm at 1” resolution^22^, though this will be lower for flatter topography where flooding predominantly occurs. Even though such errors are very low in comparison to other large-scale terrain datasets (e.g. SRTM and ASTER), they will influence the final hazard delineation. Topography is the primary control on patterns of flood inundation, more so than the governing equations of fluid dynamics^23^, meaning that even these relatively small errors in elevation may influence whether a cell is flooded or not.

Although our hazard model has gone further than other continental or global models in the representation of flood defences, it is still lacking in this area. The USACE National Levee Database is incorporated explicitly into the model, and validation studies show that doing this increases model performance markedly in defended areas^3^. The NLD, however, is estimated to be only around 30% complete^21^, meaning that a number of genuinely defended areas may be inundated in the model. Since defences are built where people and assets are more concentrated, the exposure and risk estimates in this study will be sensitive to the delineation of flood defences. Unfortunately, this limitation is not easily overcome; the incompleteness of the leading inventory of defence information (the NLD), rather than our methodology, is at fault. This study focuses on large flood events (with return period greater than 1 in 50-years) in order to minimise the effect of this, but improvements to this database and, indeed, the generation of a global one (FLOPROS shows promise at this early stage^24^) are a key requirement for future developments in large-scale flood risk analysis.

Extreme flow generation is subject to substantial uncertainty in all flood hazard models irrespective of their scale and coverage. Due to the challenges associated with modelling extreme flows in ungauged catchments (a necessary step in a continental-scale model with total coverage), we opt to use a flood frequency analysis methodology based on river gauge data rather than a rainfall-driven hydrological model. We address the issue of gauge paucity in space by adopting a regionalised flood frequency analysis (RFFA) methodology, which assumes that a gauged and ungauged catchment of similar physical characteristics (e.g. size, climatology, slope) will also have a similar flood frequency response^4^. This approach, too, is subject to high errors: global mean errors of roughly 80%, and in some cases over 300%, have been reported when comparing RFFA-derived discharges to observed ones^4^. We consider these errors to be unavoidable in models of this scale, and certainly wouldn’t be addressed by methodological change (e.g. the use of rainfall-runoff models). Only with increased observation of river flows in time and space can significant advances be made in extreme flow generation. However, our approach does mitigate much of this error by calibrating channel geometry to channel bankfull discharge as estimated by the RFFA^25^.  This step is crucial as it ensures that any biases in the RFFA are represented in the hydraulic model channel calibration (i.e. if the RFFA has a positive bias for a particular river, the estimated bankfull discharge will be larger than expected and so the channel will be deeper than expected to allow a higher conveyance). We therefore have confidence that the model developed in this study is adequate for the purpose to which it is put and that the conclusions from the analysis are robust.

**5.2 Uncertainty in exposure estimation**

Exposure estimation, at least for the present-day, is probably the least error-prone stage of this analysis. Despite being sourced from aggregate data (e.g. census-block level), the dasymetric distribution of people in the USEPA population map and assets in the FEMA National Structure Inventory is likely approaching the ceiling for accuracy of such data. There will be considerable uncertainty in the values assigned to assets, however.

Future socio-economic projections will naturally be highly uncertain. We won’t ever know for sure what the CONUS will look like in a demographic and developmental sense up to 2100. USEPA ICLUS projections, also distributed dasymetrically, probably give the most accurate and plausible view of this that is currently possible, though it should be noted that these projections offer only two feasible scenarios amongst infinite possibilities. The method by which particular land-use classes are assigned economic values, however, is another major source of uncertainty. By intersecting the NSI with the NLUD, assigning per-pixel values to individual classes and iterating this across future land-use maps, we have generated reasonable approximations to future asset values, but a number of important factors are not accounted for. In particular, homogeneity of asset value within a certain class is assumed. This means a densely built-up pixel in Los Angeles has the same economic value as one in Detroit: a somewhat unrealistic assumption. Notwithstanding these limitations, our exposure methodology is a substantial improvement on that of previous large-scale estimates and produces worthy estimates for the purposes they are intended for.

**5.3 Uncertainty in risk estimation**

Our estimates of flood risk, which is confined to potential flood damage, accumulates the wealth of aforementioned uncertainty as well as that pertaining to depth-damage functions. Estimates of present-day flood risk employ these relationships in a more realistic way than those of the future by applying a specific USACE depth-damage curve for each type of building. In this way, information such as the number of storeys a building has or whether it has a basement are accounted for. Measures of vulnerability, however, depend far more on information about the age of a building or what it is built of. Localised information is also critical: a certain depth of water would affect properties in urban New York differently to those in rural Texas. It would be prohibitively difficult to account for such characteristics in a model of this scale. Besides, it is likely that, at the aggregate scale and return periods at which it is applied, our analysis largely cancels out these local errors.

The FIA-derived generalised depth-damage curve is applied across the CONUS for future projections, meaning the type of structure is not accounted for. Such information simply isn’t available for the future, so this approach, with an aggregated relationship weighted towards more frequently flooded building types, has little scope for improvement. Nevertheless, there is still substantial value in the output of this analysis.

**6 Supplementary Figures**


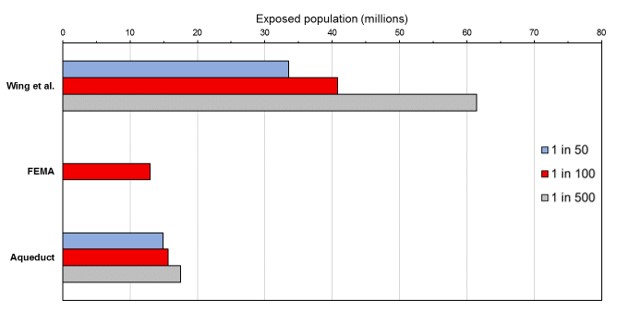


**Supplementary Figure 1.** Total population exposed to flooding in the CONUS for the present-day. As well as containing results from this analysis, the graph displays FEMA-derived and Aqueduct exposure estimates.


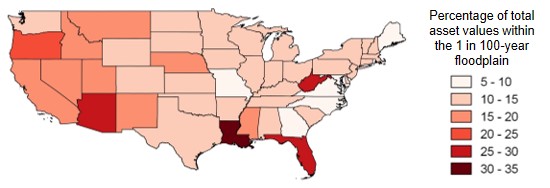


**Supplementary Figure 2.** Proportion, in terms of their economic value, of assets within the 1 in 100-year floodplain.


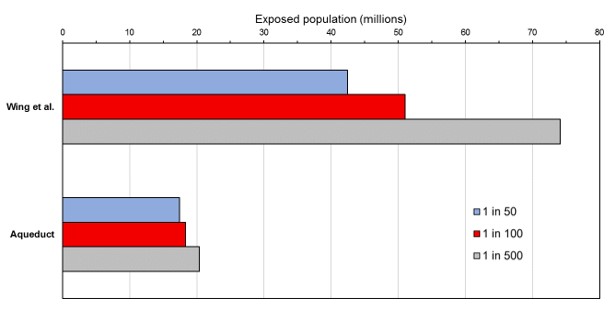


**Supplementary Figure 3.** Population exposure estimates for 2030 in CONUS under the SSP2 scenario.


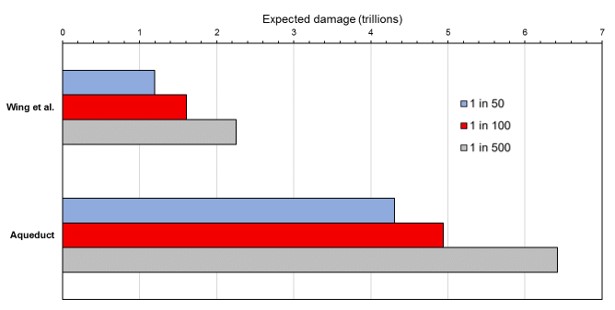


**Supplementary Figure 4.** Expected flood damages for 2030 in CONUS under the SSP2 scenario.


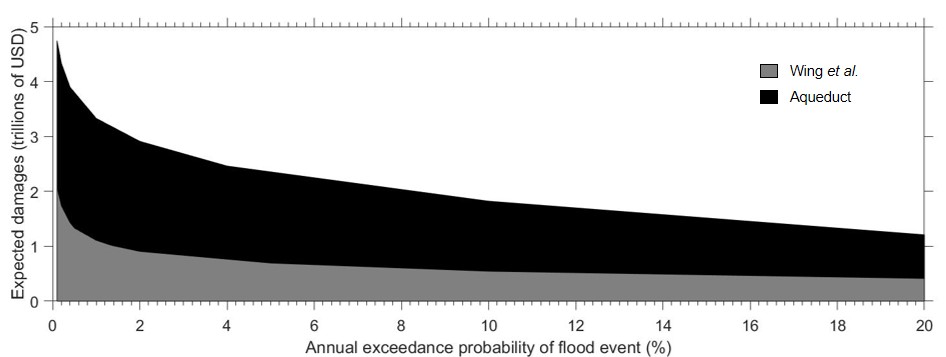


**Supplementary Figure 5.** Exceedance probability-impact curves from this analysis and with Aqueduct data.

**7 Supplementary Tables**

**Supplementary Table 1.** Present-day population-based flood exposure in the CONUS based on the analysis in this paper.

| Return Period | Exposed population | Percentage population exposure | GDP exposure (trillions of USD) | GDP exposure (% of US GDP) |
| --- | --- | --- | --- | --- |
| 1 in 50  1 in 100  1 in 500 | 33,567,281 | 10.95 | 2.39 | 12.56 |
|  | 40,817,905 | 13.31 | 2.91 | 15.31 |
|  | 61,443,761 | 20.04 | 4.40 | 23.12 |

**Supplementary Table 2.** Present-day asset exposure and risk estimates in the CONUS.

| Return Period | Area of exposed developed land (km^2^) | | Total value of exposed assets (trillions of USD) | | Expected damages (trillions of USD) | | Number of exposed assets (millions) | |  |
| --- | --- | --- | --- | --- | --- | --- | --- | --- | --- |
| 1 in 50 | | 140,657 | | 4.62 | | 0.94 | | 12.9 | |
| 1 in 100 | | 157,430 | | 5.53 | | 1.16 | | 15.4 | |
| 1 in 500 | | 203,775 | | 8.17 | | 1.86 | | 22.6 | |

**Supplementary Table 3.** Future population exposure in the CONUS according to this analysis.

| Scenario | Return Period | Exposed population | Percentage exposure |
| --- | --- | --- | --- |
| 2050 (SSP2) | 1 in 50 | 51,344,66 | 13.12 |
|  | 1 in 100 | 61,214,846 | 15.64 |
|  | 1 in 500 | 86,842,731 | 22.19 |
| 2050 (SSP5) | 1 in 50 | 61,893,832 | 13.29 |
|  | 1 in 100 | 73,409,641 | 15.76 |
|  | 1 in 500 | 103,410,662 | 22.20 |
| 2100 (SSP2) | 1 in 50 | 63,094,198 | 13.86 |
|  | 1 in 100 | 74,834,300 | 16.44 |
|  | 1 in 500 | 104,535,565 | 22.97 |
| 2100 (SSP5) | 1 in 50 | 104,633,823 | 14.34 |
|  | 1 in 100 | 122,615,504 | 16.80 |
|  | 1 in 500 | 168,454,913 | 23.08 |

**Supplementary Table 4.** Future asset exposure and risk estimates in the CONUS.

| Scenario | Return Period | | | Area of exposed developed land (km^2^) | Total value of exposed assets (trillions of USD) | | Expected damages (trillions of USD) | |
| --- | --- | --- | --- | --- | --- | --- | --- | --- |
| 2050 (SSP2) | | 1 in 50 | 174,989 | | | 6.88 | | 1.45 |
|  |  | 1 in 100 | 195,981 | | | 8.07 | | 2.06 |
|  |  | 1 in 500 | 251,702 | | | 11.29 | | 2.65 |
| 2050 (SSP5) | | 1 in 50 | 193,023 | | | 8.08 | | 1.68 |
|  |  | 1 in 100 | 216,348 | | | 9.46 | | 2.03 |
|  |  | 1 in 500 | 277,233 | | | 13.16 | | 3.06 |
| 2100 (SSP2) | | 1 in 50 | 192,417 | | | 8.34 | | 1.72 |
|  |  | 1 in 100 | 215,900 | | | 9.78 | | 2.09 |
|  |  | 1 in 500 | 276,956 | | | 13.61 | | 3.16 |
| 2100 (SSP5) | | 1 in 50 | 240,941 | | | 10.33 | | 2.72 |
|  |  | 1 in 100 | 271,106 | | | 15.52 | | 3.29 |
|  |  | 1 in 500 | 346,968 | | | 21.19 | | 4.89 |

**8 Supplementary References**

1. United Nations International Strategy for Disaster Risk Reduction. Making Development Sustainable: The Future of Disaster Risk Management. Global Assessment Report on Disaster Risk Reduction. (United Nations Office for Disaster Risk Reduction, Geneva, Switzerland, 2015).
2. Jongman, B. *et al.* Comparative flood damage model assessment: towards a European approach. *Nat. Hazards Earth Syst. Sci.* **12**, 3733-3752 (2012).
3. Wing, O. E. J. *et al.* Validation of a 30m resolution flood hazard model of the conterminous United States. *Water Resour. Res.* [UNDER REVIEW].
4. Smith, A., Sampson, C. & Bates, P. Regional flood frequency analysis at the global scale. *Water Resour. Res.* **51**, 539-553 (2015).
5. Lehner, B., Verdin, K. & Jarvis, A. New global hydrography derived from spaceborne elevation data. *Eos, Transactions, American Geophysical Union* **89**, 93-94 (2008).
6. Neal, J., Schumann, G. & Bates, P. A subgrid channel model for simulating river hydraulics and floodplain inundation over large and data sparse areas. *Water Resour. Res.* **48**, W11506 (2012).
7. Bates, P. D., Horritt, M. S. & Fewtrell, T. J. A simple inertial formulation of the shallow water equations for efficient two-dimensional flood inundation modelling. *J. Hydrol.* **387**, 33-45 (2010).
8. Morin, J. & Benyamini, Y. Rainfall infiltration into bare soils. *Water Resour. Res.* **13**, 813-817 (1977).
9. Elvidge, C. D. *et al.* Global Distribution and Density of Constructed Impervious Surfaces. *Sensors* **7**, 1962-1979 (2007).
10. Seneviratne, S. *et al*. in Managing the Risks of Extreme Events and Disasters to Advance Climate Change Adaptation (eds Field, C. B. *et al*.) 109–230 (Cambridge Univ. Press, 2012).
11. Smith, A., Freer, J., Bates, P. & Sampson, C. Comparing ensemble projections of flooding against flood estimation by continuous simulation. *J. Hydrol.* **511**, 205-219 (2014).
12. Theobald, D. M. Development and Applications of a Comprehensive Land Use Classification and Map for the US. *PLOS ONE* **9**, E94628 (2014).
13. US Environmental Protection Agency. Updates to the Demographic and Spatial Allocation Models to Produce Integrated Climate and Land Use Scenarios (ICLUS) Version 2. EPA/600/R-16/366F, National Center for Environmental Assessment, Washington, DC (2016).
14. Blessing, R., Sebastian, A. & Brody, S. D. Flood Risk Delineation in the United States: How Much Loss Are We Capturing? *Nat. Hazards Rev.* **18**, 04017002 (2017).
15. Winsemius, H. C., van Beek, L. P. H., Jongman, B., Ward, P. J. & Bouwman, A. A framework for global river flood risk assessments. *Hydrol. Earth Syst. Sci.* **17**, 1871-1892 (2013).
16. Strahler, A. N. in *Handbook of Applied Hydrology* (ed Chow, V. T.) 4-39 4-76 (McGraw-Hill, New York, 1964).
17. Trigg, M. A. *et al.* The credibility challenge for global fluvial flood risk analysis. *Environ. Res. Lett.* **11**, 094014 (2016).
18. Downton, M. W., Barnard Miller, J. Z. & Pielke, R. A. Reanalysis of U.S. National Weather Service Flood Loss Database. *Nat. Hazards Rev.* **6**, 13-22 (2005).
19. National Weather Service. Hydrologic Information Center - Flood Loss Data. http://www.nws.noaa.gov/hic/ (2015).
20. Meyer, V., Haase, D. & Scheuer, S. Flood risk assessment in european river basins—concept, methods, and challenges exemplified at the Mulde river. *Integr. Environ. Assess. Manag.* **5**, 17-26 (2009).
21. American Society of Civil Engineers. 2017 Infrastructure Report Card: Levees. https://www.infrastructurereportcard.org/cat-item/levees/ (2017).
22. Gesch, D. B., Oimoen, M. J. & Evans, G. A. Accuracy Assessment of the US Geological Survey National Elevation Dataset, and Comparison with Other Large-Area Elevation Datasets – SRTM and ASTER. https://pubs.usgs.gov/of/2014/1008/pdf/ofr2014-1008.pdf (2014).
23. Horritt, M. S. & Bates, P. D. Evaluation of 1D and 2D numerical models for predicting river flood inundation, *J. Hydrol.* **268**, 97-99 (2002).
24. Scussolini, P. *et al.* FLOPROS: an evolving global database of flood protection standards. *Nat. Hazards Earth Syst. Sci.* **16**, 1049-1061 (2016).
25. Sampson, C. C. *et al.* A high-resolution global flood hazard model. *Water Resour. Res.* **51**, 7358-7381 (2015).
